# Post-COVID-19 Syndrome Comprehensive Assessment: From Clinical Diagnosis to Imaging and Biochemical-Guided Diagnosis and Management

**DOI:** 10.3390/v15020533

**Published:** 2023-02-14

**Authors:** Michael Zhipeng Yan, Ming Yang, Ching-Lung Lai

**Affiliations:** 1 Department of Medicine, The University of Hong Kong, Queen Mary Hospital, Hong Kong SAR, China; 2 Department of Ophthalmology, The University of Hong Kong, Hong Kong SAR, China

**Keywords:** long COVID-19 syndrome, post-COVID-19 syndrome, diagnosis, assessment, review

## Abstract

The COVID-19 outbreak was first reported in 2019, causing massive morbidity and mortality. The majority of the COVID-19 patients survived and developed Post-COVID-19 Syndrome (PC19S) of varying severity. Currently, the diagnosis of PC19S is achieved through history and symptomatology that cannot be explained by an alternative diagnosis. However, the heavy reliance on subjective reporting is prone to reporting errors. Besides, there is no unified diagnostic assessment tool to classify the clinical severity of patients. This leads to significant difficulties when managing patients in terms of public resource utilization, clinical progression monitorization and rehabilitation plan formulation. This narrative review aims to review current evidence of diagnosis based on triple assessment: clinical symptomatology, biochemical analysis and imaging evidence. Further assessment tools can be developed based on triple assessment to monitor patient’s clinical progression, prognosis and intervals of monitoring. It also highlights the high-risk features of patients for closer and earlier monitoring. Rehabilitation programs and related clinical trials are evaluated; however, most of them focus on cardiorespiratory fitness and psychiatric presentations such as anxiety and depression. Further research is required to establish an objective and comprehensive assessment tool to facilitate clinical management and rehabilitation plans.

## 1. Introduction

The COVID-19 outbreak was first reported in 2019, causing massive morbidity and mortality. Though various vaccine trials have shown good safety and efficacy for different populations, vaccination does not completely prevent infection and re-infection [1,2]. Though the majority of infected patients recover, a significant proportion develop Post-COVID-19 Syndrome (PC19S, aka Long-COVID-19 Condition) of varying severity. In October 2021, the WHO published the definition of Post-COVID-19 Syndrome (PC19S) as patients with a history of probable or confirmed SARS-CoV-2 infection 3 months from the onset of COVID-19 with at least 2 months of new-onset symptoms which cannot be explained by an alternative diagnosis. Currently, there are 12 documented domains of PC19S symptoms to define its diagnosis. However, the heavy reliance on subjective reporting is prone to reporting errors. Thus, an objective diagnostic tool set is required to define the diagnosis and facilitate future treatment.

Over 40–70% of COVID-19 survivors may develop PC19S [3,4]. This has a significant impact on their quality of life, mental health and employment. The effect of PC19S may last years or longer [5]. The impact of PC19S is enormous for patients, the healthcare system and economic development [6,7]. Multidisciplinary care aimed at identifying high-risk individuals and supporting long-term rehabilitation to maximize functional capacity is required [8]. Currently, the NHS has established a three-tier service model to provide targeted individualized interventions with a specific set of outcome measures outlined in the COVID-19 Yorkshire Rehabilitation Scale [9]. Similar clinical questionnaires are under research [10]. However, there is no unified assessment tool to diagnose and quantify the severity of PC19S, leading to consequences of underdiagnosis or overdiagnosis. Medical practitioners know little about its diagnosis and rehabilitation pathways [11,12]. Thus, standardized assessment and treatment tools should be provided according to resource availability in local healthcare systems to maximally safeguard the best interests of COVID-19 survivors.

**Figure 1:**
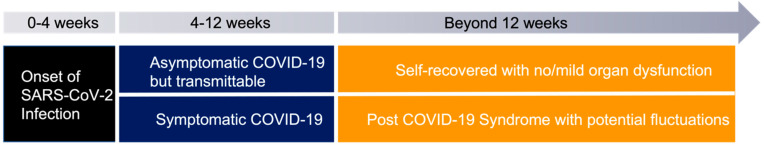
Proposed Pathogenesis model of PC19S [8,14].

Some survivors develop subclinical multi-organ impairment, which warrants further screening to guide its management [13]. Figure 1 shows the proposed pathogenesis from previous authors [8,14]. This review aims to define possible assessment tools for the diagnosis and monitoring of the progression in terms of clinical, radiological and biochemical parameters. It also defines high-risk populations for frequent follow-up, follow-up schedules and rehabilitation details for patients with PC19S.

## 2. Current Diagnosis of PC19S

**Figure 2:**
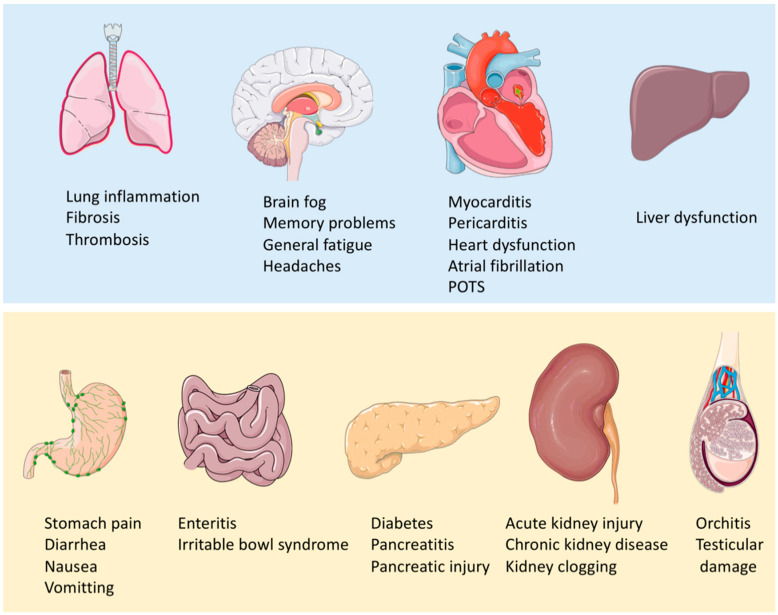
Common clinical presentations of PC19S. (Parts of the Figure was drawn and adapted using pictures from Servier Medical Art (http://smart.servier.com/) (accessed on 10 July 2022), licensed under a Creative Commons Attribution 3.0 Unported License (https://creativecommons.org/licenses/by/3.0/) (accessed on 4 February 2023).

The WHO definition of PC19S is based on symptom duration, onset and category of symptoms. Figure 2 shows the most common PC19S symptoms. Various clinical questionnaires have been proposed with reference to the incidence of clinical presentations [14]. The initial questionnaire is used to screen the severity of PC19S. The change of symptoms score is used for patients with mild symptoms for outpatient monitoring [15,16]. The initial screening tool, “the long covid Symptom and Impact Tool”, was constructed and validated in France, with a passing score of 30 for self-reported, acceptable symptom control state (95% CI, 28–33) [16]. Non-hospitalized patients are more likely to be associated with neuropsychiatric symptoms [17], while hospitalized patients have more respiratory symptoms (*p* < 0.05) [18]. Three months is set as the duration necessary for diagnosis, since over 70% of COVID-19 survivors experienced symptom resolution by 12 weeks [19].

Another validated clinical assessment tool in the UK is the “Post-Acute (Long) COVID-19 Quality of Life (PAC-19QoL) Instrument”. It identified 35/44 quality of life indicators for those with PC19S (*p* < 0.05) [20]. Several other clinical tools are used for the assessment of PC19S, such as the Short-Form-36 Health Survey (SF-36), EQ-5D-5L Assessment Tool and Health-Related Quality of Life Scale (HRQoL) [21].

It may be difficult to accurately define PC19S as different assessment schemes emerge. Exertional intolerance and dyspnea with preserved or deteriorated lung function have both been reported [22]. It is important to divide the diagnostic/monitoring pathway into asymptomatic or symptomatic pathways first, followed by a triple assessment if available (clinical, radiological and biochemical). Table 1 shows an updated review summary of PC19S on various body systems. 

## 3. Imaging Diagnosis of PC19S

Persistent lung injury has been reported in PC19S due to persistent shedding of the virus, the prothrombotic state and the ongoing inflammatory state [66]. Possible image findings include ground glass opacification, consolidation, crazy paving, vacuole sign, pulmonary nodules, lobar pneumonia, tractional bronchiectasis, vascular thickening, lung cavitations and fibrosis, predominantly at the peripheral/subpleural locations [67]. The CT severity score can be used to calculate the extent of pathological involvement in each of the 5 lobes [68,69]:

Score of 0: no involvement;
Score of 1: <5% involvement;
Score of 2: 5–25% involvement;
Score of 3: 26–50% involvement;
Score of 4: 51–75% involvement; and
Score of 5: >75% involvement.

The sum of each individual lobar score is added to give a global CT score to quantify the severity and predict the probability of PC19S [70]. A cut-off value of 7 is proposed with a sensitivity of 95.9%, specificity of 96%, positive predictive value of 95.92%, negative predictive value of 96% and accuracy of 95.96% [67].

## 4. Biochemical Diagnosis of PC19S

Understanding the inflammatory, immunological and metabolic activity biomarkers is required for the detailed assessment of PC19S [71].

Post-COVID postural tachycardia syndrome is one of the documented PC19S complications. Its diagnosis is based on clinical symptoms and an excessive orthostatic tachycardia (with a heart rate increase of >30 beats per minute in adults and 40 beats per minute in adolescents) within 10 min of upright posture in the absence of orthostatic hypotension and associated symptoms of orthostatic intolerance for at least 3 months [72,73].

In view of the various clinical manifestations of PC19S on the neurological system, some studies have investigated the effect of biomarkers on the central nervous system, such as myelin oligodendrocyte glycoprotein (MOG) and CCL-11 [74]. In patients with a neurological presentation of PC19S, reactive microglia with astrocyte reactivity, alteration of hippocampal neurogenesis, myelinated axons and oligodendrocytes have been reported in literature [75,76,77,78,79,80,81]. In view of this, several mechanisms have been proposed:

(1) Cytokine-associated immune dysregulation disrupts myelin hemostasis and plasticity, impairing hippocampal neurogenesis with neurotoxic astrocyte reactivity. This results in an impairment of neural circuit function and cognition.
(2) Anti-neural antibodies cause autoimmune encephalitis leading to immune-mediated brain injury
(3) Neuroinvasive infection
(4) COVID-19 triggers reactivation of latent virus infection, such as EBV, which can further trigger inflammation cascades and impairment of brain function
(5) Neurovascular dysfunction of the blood–brain barrier leading to proinflammatory molecules and thrombosis. Neural inflammation accounts for the disseminated brain injury
(6) Hypoxia and metabolic disturbances cause central nervous system injury

Other biochemical research on PC19S have been in progress. Persistent clotting protein pathology has been reported with increased levels of antiplasmin [82]. Microclots with inflammatory molecules have been detected in plasma samples of patients with acute COVID-19 infection and PC19S. Consideration of anticlotting therapy to support fibrinolytic system dysfunction can be considered in clinically symptomatic survivors [31]. Anticoagulation can be effective in improving outcomes as persistent systemic vascular inflammation and dysfunction by thrombosis are key factors driving various complications in PC19S [83].

Several biomarkers have been identified as having prognostic significance. PC19S is associated with long-term inflammation, thus the persistence of interleukin-6 (IL-6) and tumor necrosis factor-alpha have been shown to be of prognostic value [84,85]. The magnitude of early CD4+ T cell immune responses correlate with the severity of initial infection; thus, it can be used as a tracker of PC19S severity [85]. Inflammatory dysbiosis of the oral microbiome is associated with the duration of symptoms of PC19S [86]. Thyroid dysfunction has been observed in PC19S patients. The presence of anti-thyroid peroxidase (Anti-TPO) is a positive prognostic finding for symptom resolution in earlier stage [87].

Detection of superantigens is a proposed hypothesis requiring validation. Persistent viral RNA shedding by superantigens could overstimulate the anti-virus immune response and induce negative feedback loops [88]. Immunologically, reduced CD4+ and CD8+ memory cells are associated with PC19S. Persistent T-cell perturbations up to several months are common in mild COVID-19 survivors [89]. Immunomodulation by mesenchymal cells could be a treatment option for these patients due to their immunomodulatory and regenerative properties [90].

Biochemically, an elevated taurine and reduced glutamine/glutamate ratio in plasma samples (*p* < 0.05) have been found in patients with PC19S, indicating possible liver and muscle damage and the related energy-demanding process of recovery and tissue repair [40]. Asymptomatic patients during follow-up display a spectrum of multiple biochemical pathophysiology. The metabolic phenotyping approach for multisystem functional assessment has been recommended [91].

The association between gut microbiota disruption and PC19S severity is under investigation [71].

## 5. Prognosis of PC19S Tools

Ongoing research on PC19S prognosis has aroused interest in the field. Table 2 shows a summary of useful investigation tools for patients with PC19S. A Bangladesh cohort study showed a persistence of PC19S symptoms at 9 months ranging from 16.1% to 50% [92,93]. The mean duration of PC19S symptoms was 21.8 weeks and the length of PC19S was projected with a structural equation based on age, gender, smoking and prior functional limitations [92]. Table 3 shows a summary of the clinical assessment of the prognosis and severity of PC19S in various studies in terms of triple assessment: clinical, biochemical and radiological.

## 6. Interval of Monitoring

**Figure 3:**
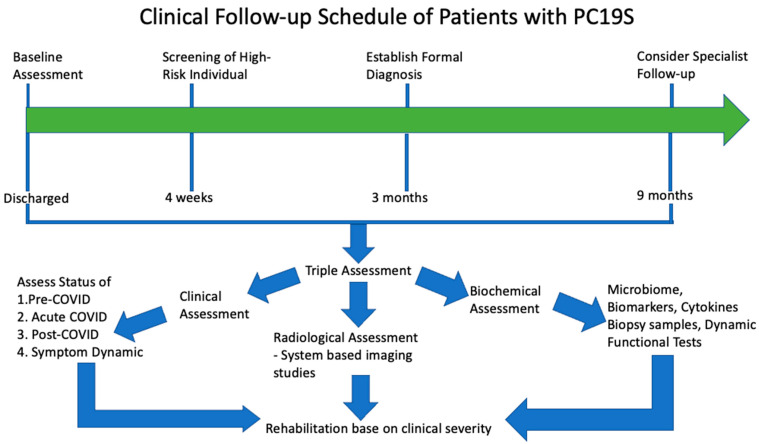
Clinical Follow-up Schedule of PC19S. Patients are recommended to have an initial clinical assessment at the time of discharge and follow-up for at least 3 months for persistent symptoms/patients with high-risk factors. Patients with persistence/progression of symptoms beyond 9 months should consider specialist referral as most symptoms disappear by 9 months. Triple assessment is recommended based on clinical, radiological and biochemical assessment for a comprehensive review. Rehabilitation programs are provided to patients, stratified by clinical severity to optimize resource usage and rehabilitation progress [8].

Figure 3 shows the clinical follow-up schedule suggested according to the summary of existing evidence. Table 4 shows the persistence of the most common symptoms across different time periods after initial COVID-19 infection. The order of first ranked symptoms varies in different studies but the top 3 ranked problems are similar: ongoing respiratory problems, persistent fatigue and cognitive deficit (brain fog) [3,102,127]. The initial follow-up schedule should be set at 28 days, 3 months and 9 months. Further follow-up sessions can be arranged if there is a progression of symptoms or abnormal imaging or laboratory findings. Screening should be done based on clinical suspicion. COVID-19-associated acute kidney injury is associated with a good prognosis: over 70% can live without renal replacement therapy and over half with complete resolution of acute kidney injury [128].

Hospital admission (odd ratio 2.28) and persistence of symptoms at day 28 (odd ratio 2.21) and month 9 (odd ratio 5.16) are independent predictors of suboptimal physical health and psychological stress [130]. However, another 9-month follow-up study showed no association between acute COVID-19 infection severity state and the number of PC19S symptoms and cognitive dysfunction [131].

There is no increase in inflammatory markers in blood tests at the subacute stage, and no association of the symptoms to biochemical parameters [132]. COVID-19-related persistent symptoms improve over time, but neurological symptoms last longer than other symptoms [98].

A 9-month grace period should be seen as a cut-off interval for top-up rehabilitation services since most PC19S symptoms should decrease after 9 months, while fatigue and somnipathy can persist for over a year [133].

## 7. Features of High-Risk Individuals for Screening

Some common features of high-risk individuals include a history of hospitalization, concomitant active cancer, medical comorbidities, use of supplemental oxygen and ventilatory support [5,67,134,135,136]. Review of clinical profiles before discharge and planning of rehabilitation schedules are recommended for hospitalized patients. These should include: patients aged over 50 (odd ratio 8.5, 95% CI 1.9–38, *p* = 0.01), longer duration of hospital stay (odd ratio 5.5, 95% CI 1.5–21, *p* = 0.01), presence of acute respiratory distress syndrome during the acute phase (odd ratio 13, 95% CI 3.3–55, *p* < 0.001), non-invasive ventilation (odd ratio 6.3, 95% CI 1.3–30, *p* = 0.02) and initial CT score more than 18 (odd ratio 4.2, 95% CI 1.2–14, *p* = 0.02) [137]. One multicenter prospective cohort study shows a higher propensity of PC19S in patients with previous lung disease and tobacco consumption [136].

PC19S has also been reported in non-hospitalized, asymptomatic patients [138]. However, continued interval monitoring for PC19S should be adopted before aggressive treatment since the longitudinal study in Faroe Islands showed symptom resolution took several months [139].

Another more recent study has shown that the number of initial symptoms is more related to PC19S than the severity of the acute infection [140].

Physicians in charge of pediatric populations should also look out for PC19S as it has been widely reported in various literature in children who demonstrate similar symptoms as adults, with an estimated incidence of over 40% [141,142,143]. Various clinical presentations have been documented such as myocarditis, postural tachycardia syndrome, mast cell disorder and mast cell activation [41,144,145,146]. Patients with these complications should be screened and followed-up every 4 weeks or sooner for close monitoring.

PC19S is common among people living with HIV and moderate to severe acute COVID-19 illness. Over 20% reported PC19S at a median follow-up of 6 months, with the most prevalent symptoms being asthenia (80%), shortness of breath (50%) and recurrent headache (25%) [147]. Integrated assessment of PC19S and anti-retroviral therapy clinical visits should be considered [148].

## 8. Persistent and Clinically Significant Symptoms to Address

Most PC19S symptoms improve over time, particularly after 9 months. A significant proportion of patients reported persistent neuropsychiatric symptoms, which indicates a bad prognosis [133]. Brain fog has been reported frequently as a notable symptom of PC19S. There is an association of brain fog with abnormal FDG-PET findings, highlighted by a hypometabolic cingulate cortex [115]. FDG-PET imaging may also show hypometabolism in the parahippocampal gyrus, cerebellum, brainstem, olfactory gyrus, temporal lobe and thalamus, which corresponds to patients with persistent anosmia/ageusia, fatigue and vascular uptake (uncorrected *p* < 0.005) [149,150]. This corresponds to cerebral hypoxia, leading to compromised neuronal cell energy metabolism and, eventually, mitochondrial dysfunction [151]. The hypoxic condition favors persistent viral shedding and compromised cognitive change, which induces brain fog and related behavioral changes [151]. The UK Biobank MRI brain cohort study showed a greater reduction in grey matter thickness and tissue damage in the orbitofrontal cortex, parahippocampal gyrus and primary olfactory area at 4 months after infection, leading to multiple clinical presentations such as fatigue, olfactory dysfunction and brain fog [152]. Similar functional changes have been validated whole brain functional analyses performed at 11 months [103]. Another long-term follow-up study of dynamic brain changes in patients who recovered from COVID-19 without neurological manifestation showed there is a co-existence of recoverable and long-term unrecoverable changes after 10 months [153]. Cognitive decline in some patients may spontaneously improve over time [154].

Clinically, the assessment can be done using various scales such as the personality assessment inventory to assess somatic preoccupation and depression [116]. In patients with persistent neurocognitive symptoms, ANA titer elevations should be checked since autoimmunity could be a cofactor in the etiology of PC19S [155]. In patients with baseline motor dysfunction, PC19S with worsening motor symptoms requires a high dosage of levodopa for disease management [156].

## 9. Rehabilitation Program

**Figure 4:**
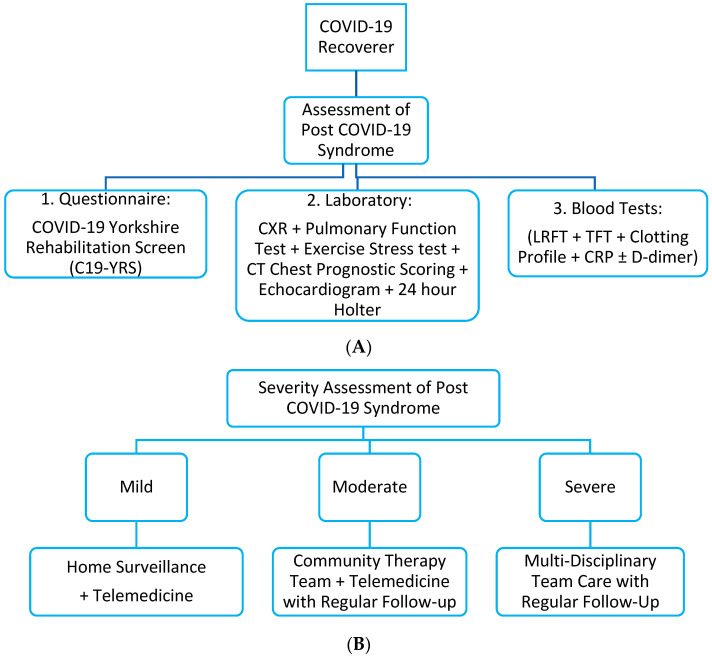
Recommended rehabilitation model for patients with Post-COVID-19 Syndrome [8], as previously suggested by other authors. However, the key point lies in the severity assessment of Post-COVID-19 Syndrome and its classification. The limitation with this classification system is that there has not been a unified classification system for clinical severity. (**A**) Basic screening assessment of Post-COVID-19 Syndrome with prognostic scoring. (**B**) Recommended rehabilitation model based on initial assessment. CXR: Chest X-Ray. LRFT: Liver Renal Function Test. CRP: C-Reactive Protein. CT: Computed Tomography.

The goals of rehabilitation should be individualized based on cardiorespiratory fitness, muscle function, bone and joint health, functional capacity and quality of life [157]. In terms of rehabilitation progress, clinically significant pulmonary function improvement may take a longer time, while muscle strength, walking capacity, sit-to-stand performance and quality of life usually improve earlier [158]. The initial lower physical composite score from the Short-Form Survey (SF-12) is associated with a prolonged recovery [99]. Figure 4 shows a suggested rehabilitation framework for patients with PC19S, and Table 5 shows clinicians’ global research progress related to rehabilitation programs for PC19S.

Moderate impairment of physical capacity caused by muscle deconditioning has been observed in post-COVID-19 patients. Immobilization during its acute episodes further aggravates muscle deconditioning. Sarcopenia incidence is high, complicated by physical inactivity, lockdown, quarantine or acute hospitalization with bed rest [179]. Several small-scale studies have highlighted the importance of cardiopulmonary rehabilitation [180,181,182,183]. A recent clinical trial, the “COVID-Rehab Study”, recruited post-COVID-19 survivors for rehabilitation. This study equips them with aerobic exercise, muscle strengthening and respiratory exercise 3 times per week over 8 weeks with the aim to measure the effect of exercise on various parameters in patients with PC19S, such as cardiorespiratory fitness, neuropsychological assessment, physical strength and functional capacity of body balance and inflammatory markers [167]. The use of oxygen–ozone autohemotherapy has been shown to induce a significant reduction in chronic fatigue in a trial of 100 patients (H = 148.4786, *p* < 0.01) [136]. The underlying reason for exercise rehabilitation for PC19S is related to the mediation of the anti-inflammatory response, the support of brain homeostasis and the increase in insulin sensitivity to counteract the neuropsychiatric and endocrine sequelae of PC19S [184].

Optimization of comorbidities is a key to rehabilitation. Diabetes mellitus and underlying cardiovascular risk factors play vital roles in disease management [149]. Strict control of diabetes with an optimization of comorbidities, supervised rehabilitation with physical exercise and an optimization of nutrition is recommended for the management of PC19S [185,186]. There is some evidence that the adoption of a plant-based diet could benefit PC19S management due to a reduction of pro-inflammatory mediators [187].

A 7-week psychologist-led interdisciplinary virtual rehabilitation program showed significant improvements in health-related quality of life in terms of mobility, self-care, activities of daily living, pain/discomfort and anxiety/depression [188]. Another 6-week interdisciplinary individualized pulmonary rehabilitation program showed promising findings including an improvement in the 6-min walking distance, pulmonary function test, post-COVID-19 functional status, Borg dyspnea scale, Fatigue Assessment Scale and quality of life [168]. Recent studies have also recommended the role of erectile dysfunction as a biomarker for systemic complications of PC19S, and rehabilitation of sexual health should also be included [189].

A multidisciplinary model showed effective improvement of self-reported symptom scores [190]. Primary care physicians are crucial to curb preventable adverse events through the use of clinical questionnaires, imaging assessments and serial biomarker monitoring to detect early complications and provide timely treatment [191].

In view of the growing number of PC19S cases globally, early identification of affected individuals to provide appropriate and efficient treatment is required [192]. The use of telemedicine for rehabilitation is suitable for long-term PC19S monitoring [193]. It provides a reliable and flexible assessment of patient’s health status through remote monitoring, and identification of disorders and complications through long-term monitoring of health parameters. It also reduces patient’s anxiety toward PC19S. Mental health video consultations is a useful alternative to screen patients with PC19S-related psychiatric symptoms in primary care [194]. Peer support groups are another trend which fills professional care gaps, raises societal awareness and increases public engagement [195].

Neuropsychiatric complaints are a major reason for consultation after 9 months. Several models have been proposed for these patients [196] such as the UT Southwestern Medical Centre COVIER Recover Program, UT Health San Antonio Program, VA Greater Los Angeles Healthcare System, Hennepin Healthcare and University of Florida Model. All of these are designed based on population characteristics, complication prevalence at different periods and resource availability. Baseline assessment for medical need is the first step in order to differentiate those who require self-management, acute hospitalization, community rehabilitation and more advanced rehabilitation based on their physical, cognitive and behavioral symptoms, and the relevant assessment scoring system. Multidisciplinary rehabilitation teams and specialist care will be provided for those with moderate/severe complications.

In view of long-lasting symptoms with a strong probability of progression in PC19S, various clinical studies focusing on symptom trajectory and rehabilitation progress have been published/are in progress. A brief summary has been documented in Table 5. Most of these studies primarily focus on cardiorespiratory fitness and neuropsychiatric symptoms such as chronic fatigue syndrome, depression, anxiety and functional independence measures. Yet, there is no unified rehabilitation program that has shown superior efficacy compared to others since individualized programs may be more efficacious than group training in terms of cardiorespiratory outcomes [82].

PC19S-related fatigue is a common symptom reported in literature. Rehabilitation has shown great efficacy in reducing fatigue. A systematic review and meta-analysis showed that over 80% patients reported no persistent fatigue after rehabilitation [197]. Several studies worldwide have shown that rehabilitation results in a statistically significant improvement in walking tests and the Borg Dyspnea Scale [166,198,199,200,201,202].

## 10. Role of Vaccination in PC19S Rehabilitation

Vaccination confers partial protection from PC19S. An earlier study showed vaccination may contribute to a reduction in the population health burden of PC19S: the first dose reduces the odds of PC19S by 12.8% and the second dose reduces the odds by 8.8 (*p* < 0.01) [203]. However, in patients who already have PC19S, a delay of vaccination should be considered since a study showed that increases in antibody titers in patients with diagnosed PC19S might significantly worsen clinical symptoms [204].

The COVID-19 vaccine reduces PC19S sequelae by 15% (HR 0.85, 95% CI 0.82–0.89), including cardiovascular, hematological, gastrointestinal, mental health, musculoskeletal and neuropsychiatric sequelae [205]. Owing to the, at best partial, protection of PC19S resulting from COVID-19 vaccines, it is not an optimal strategy to reduce PC19S sequelae [205]. Vaccinated individuals are recommended to take effective infection control measures and booster vaccinations since vaccinated individuals reported less frequent and shorter PC19S symptoms in the British study [206].

## 11. Future Trends

In spite of the enormous population with PC19S, only 7% received PC19S care in the public sector and among those requiring additional help for PC19S, over half prefer assistance by digital health interventions (DHI) [207]. DHI-related research is under progress worldwide to understand its application in the remote monitoring of PC19S as it is seen as an economical rehabilitation model [208,209,210]. Future directions should include restructuring of PC19S rehabilitation services including both face-to-face consultations and digital interventions to allow flexibility.

The pathophysiology, prognostication scoring system and correlations with underlying comorbidities in different diseased populations are currently under investigation [211]. Mapping of clinical symptoms to the Human Phenotype Ontology for unification of PC19S reporting has been recommended to improve the stratification, diagnosis, treatment and scientific research of PC19S [212]. Different diagnostic criteria have been proposed, but further research into imaging and biochemical diagnosis is required for a definitive approach [213].

Multidisciplinary collaboration with a clear pathway for the diagnosis and management of PC19S is required globally in view of the surging number of patients with PC19S. A clear and integrated care system consisting of holistic healthcare pathways, detection of system-specific complications, management of mild symptoms and tailored rehabilitation services should be planned for in the future.

## Figures and Tables

**Table 1:** Summary of PC19S effect on various systems [8], an updated review.

Organ System	Main Diagnosis	Features	Possible Mechanisms	Prognosis	References
Respiratory	Acute respiratory distress syndrome (ARDS)	Extensive bilateral diffuse alveolar damage with cellular fibromyxoid exudates; desquamation of pneumocytes and hyaline membrane formations; diffusion impairment and pulmonary fibrosis	SARS-CoV-2 spike S1 domain protein binding to ACE2 receptor; post-acute respiratory distress syndrome, fibrosis with diffuse alveolar damage, upregulated inflammatory responses in the respiratory mucosa	Pulmonary function deficit 6 months after infection; extensive diffuse impairment; long-term in-situ thrombosis	[23,24,25,26,27,28,29,30,31,32,33,34,35]
Cardiovascular	Endothelitis; micro-thrombosis, capillary damage; hypercoagulability; microangiopathy; thromboembolism; myocarditis; atrial fibrillation; supraventricular tachycardia, new onset hypertension, poor control of hypertension	Increased target-to-blood pool ratio; capillary disturbance; impaired oxygen diffusion	Cytokine storm and macrophage activating syndrome-caused endothelial dysfunction, direct viral toxicity, thrombosis, vasculitis, autoimmune response	Majority (81%) of the COVID-19 myocarditis patients survived the acute episode; ongoing subclinical myocarditis may evolve into myocardial dysfunction and sudden cardiac death	[36,37,38,39,40,41]
Haematological	Thromboembolism	Elevated convalescent D-dimer and C-reactive protein levels; persistently increased biomarkers of inflammation	N/A	Prognostic biomarkers for monitoring the clinical progression of post-COVID-19 patients need to be investigated	[42,43,44]
Urinary	Acute kidney injury; renal failure;	Declined glomerular filtration rate (eGFR); kidney infarction	High abundance pf ACE2 expression in kidneys	Significant risks of mortality and morbidity	[45,46,47,48]
Digestive	Gastrointestinal impairment and dysfunction; hepatitic and cholestatic liver injury; pancreatic injury	Diffuse bowel damage; Enterocyte desquamation, edema, small bowel dilation, lymphocyte infiltration and mesenteric node hemorrhage and necrosis	Rich in ACE2 and furin expression; fecal–oral transmission; plasma cells and lymphocytic infiltrations into the lamina propria of intestinal tissues	The liver enzymes remained persistently elevated 14 days after discharge, while the liver functions in the majority of survivors normalized 2 months after hospital discharge	[49,50,51,52,53,54,55]
Neurological	Mood changes; cognitive difficulties; headache; fatigue; dizziness; memory loss; confusion; attention deficit	Hypoxic injury; microbleeding; neuronal inflammations	Blood vessel damage, impaired oxygen supply, viral infiltration into the central nervous system and inflammatory cytokine-mediated cellular damage; indirect T-cell and microglia damage in the brain, similar to strokes and neuroinflammatory diseases	Over 40% of survivors without prior psychiatric conditions lived with depression within 90 days of recovery from severe COVID-19-associated respiratory failure, and 70% of them did not receive treatment for depression	[56,57,58,59,60,61]
Metabolic	Hyperglycemia without diabetic mellitus; new-onset diabetic mellitus; starvation ketoacidosis	High blood glucose level; impaired glucose metabolism	Intruding pancreatic β-islet cells, triggering autoimmune responses because of the exposure of the antigen from damaged islet cells	Long-term treatment of diabetic mellitus is needed	[62,63,64,65]

**Table 2:** Useful investigation tools for patients with Post-COVID-19 Syndrome [8].

Organ System	Main Diagnosis	Features	Useful Investigation Tools	Abnormalities to Look For	References
Respiratory	Acute respiratory distress syndrome (ARDS), respiratory failure, pulmonary embolism/thromboembolism, pneumonia, pulmonary vascular damage	Extensive bilateral diffuse alveolar damage with cellular fibromyxoid exudates; desquamation of pneumocytes and hyaline membrane formations; diffusion impairment	Pulmonary function tests, high resolution CT, histology; pulmonary angiopathy	Restrictive pulmonary function test; impaired gas transfer; reduced total lung capacity; fibrotic features on imaging; diffuse alveolar damage on histology; pulmonary vasculature	[24,27,29,31,32,94,95,96,97]
Cardiovascular	Endothelitis; microthrombosis, capillary damage; hypercoagulability; microangiopathy; thromboembolism; myocarditis; atrial fibrillation; supraventricular tachycardia, myocardial hypertrophy, atherosclerosis, myocardial fibrosis, new onset hypertension	Increased target-to-blood pool ratio; capillary disturbance; impaired oxygen diffusion	Electrocardiogram; Echocardiography; coronary angiography and cardiac catheterization; chest X-Ray; electron-beam computed tomography; cardiac MRI	Microcirculation disturbance; increased target-to-blood pool ratio; impaired oxygen diffusion; myocardial inflammation; rhythmic abnormality, lower ventricular ejection fraction, ventricular volume, gadolinium enhancement	[98,99,100,101,102,103]
Hematological	Thromboembolism, vascular hemostasis, blood coagulation	Elevated convalescent D-dimer and C-reactive protein levels; persistently increased biomarkers of inflammation	Venipuncture for blood tests of D-dimer and C-reactive protein; duplex ultrasound for lower limb clots; CT-pulmonary angiogram for pulmonary embolism; electrocardiogram, echocardiography, coronary angiography and cardiac catheterization for evidence of cardiac or pulmonary embolism	Thrombocytopenia; blood cell abnormalities	[96,104,105]
Urinary	Acute kidney injury; renal failure	Declined glomerular filtration rate (eGFR); kidney infarction	Urine analysis; glomerular filtration rate; ultrasound scanning; MRA; renal biopsy	Early recognition of kidney involvement; kidney injury; renal infarction	[46,47,106]
Digestive	Gastrointestinal impairment and dysfunction; hepatitic and cholestatic liver injury; pancreatic injury, abdominal pain, anorexia, acid reflux, gastrointestinal hemorrhage	Diffuse bowel damage; Enterocyte desquamation, edema, small bowel dilation, lymphocyte infiltration and mesenteric node hemorrhage and necrosis	Barium beefsteak meal; colorectal transit study; computed tomography scan (CT or CAT scan); defecography; lower gastrointestinal series; MRI; magnetic resonance cholangiopancreatography (MRCP); oropharyngeal motility (swallowing) study	Bowel damage; high fecal calprotectin level; gastrointestinal dysfunction; liver injury; pancreatic injury; hyperamylasemia	[49,50,51,52,53,54,55]
Neurological	Mood changes; cognitive difficulties; headache; fatigue; dizziness; memory loss; confusion; attention deficit; confusion; neuropathic pain; dizziness; convulsions; stress; depression; anxiety	Hypoxic injury; microbleedings; neuronal inflammations.	CT scan; electroencephalogram; MRI; electrodiagnostic tests such as electromyography (EMG) and nerve conduction velocity (NCV); positron emission tomography (PET); arteriogram (angiogram); lumbar puncture; evoked potentials	Neurological symptoms; ischemic damages to cerebral white matter; blood vessel damage; hypoxic injury, microbleeding and neuronal inflammation in different areas of brains; brain hypometabolism	[56,57,58,59,60,61]
Metabolic	Hyperglycemia without diabetic mellitus; new-onset diabetic mellitus; starvation ketoacidosis	High blood glucose level; impaired glucose metabolism	Blood tests for blood glucose and HbA1c level; plasma amino acid analysis; plasma carnitine level; plasma acylcarnitine profile; plasma C-peptide level, urine organic acid analysis; urine and plasma ketone analysis	Impaired glucose metabolism; increased ketone bodies	[62,63,107]

**Table 3:** Summary of PC19S clinical, imaging and biochemical markers for diagnosis and prognosis in various studies (Toolbox).

Organ System	Clinical Test	Imaging/Functional Test	Biochemical	References
Respiratory	Swiss COVID Lung Study Group 13-questions, Pody plethysmography, King’s Brief Interstitial Lung Disease (K-BILD) and Transition Dyspnea Index (TDI), Chester Step Test	Spirometry (VO2 max), respiratory muscle strength, 6-min walking test, diffusion capacity of lung, high resolution CT histology, pulmonary angiopathy	Th1 and Th2 inflammatory responses, CCL-24 level, IL-6, TNF-alpha, CCL-11, IL-33, CCL-17	[13,14,24,29,31,32,35,94,95,108,109,110,111]
Cardiovascular	Exercise stress testing, clinical questionnaire, Incremental Shuttle Walk Test (ISWT), Short Performance Physical Battery (SPPB), self-reported health-related quality of life (HR-QoL)	Electrocardiogram, chest X-Ray, echocardiogram cardiac MRI, coronary angiography, cardiac catheterization, electron beam computed tomography, cardiorespiratory fitness by peak oxygen intake	Troponin, HT-pro-BNP, d-dimer, endomyocardial biopsy (active lymphocytic infiltration)	[36,37,38,39,40,96,110,112,113,114]
Neuropsychological	MOCA, Hopkins Verbal Learning Test (verbal memory), Digit span (short-term and oral memory), Trail-Making Test (executive function), Phonological and semantic verbal fluency test (language and executive function), Personality Assessment Inventory, GAD-7 Questionnaire, Perceived Stress Scale, Hamilton Depression Scale, Hamilton Anxiety Score	CT Scan, MRI, electrodiagnostic tests such as electromyography (EMG) and nerve conduction velocity (NCV); positron emission tomography (PET); arteriogram (angiogram), lumbar puncture, evoked potential, FDG-PET imaging	Inflammatory markers, astrocyte activation, tryptophan metabolism, mannose-binding lectin deficiency	[56,57,58,59,60,61,115,116,117,118,119,120,121]
Gastrointestinal	Mini-Nutritional Assessment	Colorectal transit study, computed tomography scan, MRI, MRCP, oropharyngeal motility study	Barium breakfast meal, fecal microbiota study, microbiome composition and function, plasma serotonin, tryptophan metabolism	[49,50,51,52,53,54,55,122,123]
Musculoskeletal	Sit-to-Stand Test, Timed Up-and-Go Test 6-min walking test, 2-min step test, PROMIS Global-10 Assessment, PHQ-9, Grip Strength	Dual-Energy X-Ray Absorptiometry (DEXA)	Subcutaneous adipose ACE-2 mRNA expression, insulin sensitivity, free fatty acid, Neuropilin-1	[114,122,124,125]
Hematological	NA	Duplex ultrasound of lower limbs, CT pulmonary angiogram, electrocardiogram, echocardiogram, coronary angiography, cardiac catheterization	CBC, clotting profile (PT, APTT, INR, D-dimer), CRP, TNF-alpha, IL-1, IL-6, IL-10, LDH, uric acid, CRP, albumin, myeloperoxidase	[104,105,126]
General/Metabolic	Self-reported questionnaire SF-36stress (state–trait anxiety inventory questionnaire, perceived stress scale questionnaire) Sleep quality (Pittsburgh sleep quality index questionnaire) Post-COVID-19 symptom questionnaire EuroQoL-5-dimension (EQ-5D index), PHQ-19, GAD-7, Activity-Specific Balance Confidence (ABC scale)	NA	Blood glucose, HbA1c, plasma amino acid analysis, plasma carnitine level, plasma acylcarnitine profile, plasma C-peptide level, urine organic acid analysis, urine and plasma ketone analysis	[21,62,63,98,99,100,107,120]
Reproductive	Aging Male’s Symptom Score 44	Ultrasound scanning, magnetic resonance angiography, renal biopsy	Low free testosterone level (<5.5 pg/mL), urine analysis, glomerular filtration rate	[10,46,47,101,106]

**Table 4:** Prevalence of PC19S symptoms at different follow-up periods [3,102,127,129].

	3–6 Months	6–9 Months	9–12 Months	>12 Months
1st ranked symptoms	Fatigue (32%)	Effort intolerance(45%)	Fatigue (37%)	Fatigue (41%)
2nd ranked symptoms	Dyspnea (25%)	Fatigue (36%)	Dyspnea (21%)	Dyspnea (31%)
3rd ranked symptoms	Sleep disorder (24%)	Sleep disorder (29%)	Not reported	Sleep disorder (30%)
4th ranked symptoms	Difficulty concentrating (22%)	Dyspnea (25%)	Not reported	Myalgia (22%)

**Table 5:** Rehabilitation programs and clinical studies showed efficacy for clinical improvement of PC19S.

Rehabilitation Focus	Type of Study and Reference	Country	Measurement	Findings
Respiratory	RCT: Inspiratory Muscle Training (IMT) [111]	UK	King’s Brief Interstitial Lung Disease (K-BILD), Transition Dyspnea Index (TDI), Respiratory Muscle Strength, Chester Step Test	IMT improves breastlessness and chest symptoms in K-BILD domains and demonstrates clinically meaningful improvements in the TDI breathlessness measure, muscle strength and aerobic fitness
Respiratory	Cohort Study: Your COVID Recovery Digital Program [159]	UK	Questionnaire (EuroQoL 5-Dimension 5-Level, EQ-5D-5L), Chronic Obstructive Pulmonary Disease Assessment Test (CAT)	Statistically significant increase in EQ-5D-5L visual analogue scale (VAS) score, pre = 48.8, post = 59.9; *p* < 0.01) CAT score improved significantly (pre = 19.8, post = 15.6; *p* < 0.01)
Neuropsychiatric	RCT: “Singing for Lung Health (SLH): Improving Experiences of Lung Disease Trial” [100]	UK	Short-Form 36 Health Survey, COPD Assessment Test Score (CAT), Dyspnea-12 breathless score, PHQ-9 score for depression, GAD-7 for anxiety, ABC scale for balance confidence	Possible improvements in depression (treatment effect −4.78 PHQ-9 points, *p* < 0.05, MCID 5) and balance confidence (treatment effect +17.21 ABC scale points, *p* = 0.04, MCID 14.2)
Neuropsychiatric and Respiratory	RCT: ENO Breathe [160]	UK	Short-Form 36 Health Survey, Mental Health Composite (MHC) and Physical Health Composite (PHC) scores. CAT score, VAS breathlessness score	Improvement in MHC score (regression coefficient 2.42, 95% CI 0.03–4.80, *p* = 0.047). Improvement in VAS breathlessness score -10.48 (95% CI −17.23 to −3.73, *p* < 0.05)
Cardiovascular Respiratory	Prospective Cohort: 12-week cardiac telerehabilitation program [161]	Czech Republic	Cardiorespiratory Fitness (CRF) and Health-Related Quality of Life (HRQL)	Higher average peak O2 uptake in home-based telerehabilitation group vs. center-based rehabilitation group (25.5 vs. 23.6, *p* < 0.05)
Cardiovascular Respiratory Musculoskeletal	RCT: Multicomponent concurrent training for 8 weeks [162]	Spain	VO2 max uptake, sit-to-stand, bench press, half squat, depression scale, functional status	Results after training: VO2 max: +5.7% Sit-to-stand: −22.7% fatigue Bench press: +6.3% Half squat: +16.9% All with *p*-value < 0.05 compared to controls
Neuropsychiatric Musculoskeletal	RCT: Low intensity vs. high intensity concurrent training [163]	Saudi Arabia	Clinical parameters: Muscle strength and muscle mass Psychological: Quality of life scales	No significant difference between muscle mass in both groupsBetter hand grip strength and quality of life in low-intensity training group
Respiratory	RCT: Inspiratory Muscle Training (IMT) vs. IMT + Manual Diaphragm Release (MDR) [164]	Egypt	Maximum static inspiratory pressure, peripheral arterial blood pressure, MRC scale for dyspnea, Fatigue Severity Scale, Serum Lactate Level, 6-min walk test distance	Significant improvement in all outcome measures in favor of the study group (*p* < 0.001)
Cardiovascular	Prospective Cohort: FAITH trial [165]	USA	Ideal cardiovascular health based on AHA Life’s Simple 7 (LS7)	Use of mobile health tools successfully increased ideal cardiovascular health
Cardiovascular Respiratory Musculoskeletal	Prospective Cohort: 6-week, twice supervised rehabilitation program [166]	UK	Incremental Shuttle Walking Endurance Shuttle Walking Test	Improved by 112m on incremental shuttle walking test and 544 s on endurance shuttle walking test
Cardiovascular Respiratory	RCT: COVID-Rehab Study [167]	Canada	Cardiorespiratory fitness (VO2 max uptake), functional capacity, quality of life, perceived stress, spirometry test, coagulation and inflammatory stress profile, neuropsychiatric tests	Individualized rehabilitation training programs including aerobic exercise; muscle strengthening and specific breathing exercises enabled better recovery outcomes
Respiratory	Prospective Cohort: Outpatient Pulmonary Rehabilitation Program [168]	Austria	6-Minute Walking Test (6MWD) Post-COVID-19 Functional Status Scale (PCFS), Borg Dyspnea Scale, Fatigue Assessment Scale, Quality of Life, Pulmonary Function Test	Improved 6MWD by 62.9 m (*p* < 0.001) and 1 grade improvement of PCFS, dyspnea, fatigue and quality of life (*p* < 0.01) Improved pulmonary function parameters (FEV1, lung diffusion capacity and inspiratory muscle pressure)
Respiratory	Prospective Cohort16 concurrent training sessions [169]	Brazil	6-Minute Walking Test, Spirometry, Respiratory Muscle Strength, Dynamometry, COPD Assessment Test, PCFS	Improved functional capacity, lung function and respiratory muscle strength
Cardiovascular Respiratory Musculoskeletal	Prospective Cohort: Respiratory Rehabilitation Program [170]	Slovenia	10-metre walking test, 6 min walking test, de Morton Mobility Index	Clinically important and statistically significant improvements found in all outcome measures, with gains in motor functional independence measures
Neuropsychiatric Cardiovascular Respiratory	Prospective Cohort: Multidisciplinary rehabilitation program [171]	Spain	Motor functional independence, upper and lower limb functionality, impact of fatigue on daily activities, respiratory muscle strength, cognitive performance, quality of life	Eight weeks of multidisciplinary rehabilitation significantly reduced disability and improve functionality and quality of life, with improvement in all outcome measures
Cardiovascular Respiratory Neuropsychiatric	RCT: Children land-based vs. water-based rehabilitation program [172]	Poland	Exercise capacity, fatigue, health-related quality of life, pulmonary function	In Progress
Cardiovascular Respiratory Neuropsychiatric	RCT: Telerehabilitation vs. home exercise program following discharge from ICU [173]	Turkey	Walking distance, muscle strength, muscle endurance, quality of life, physical activity level and perceived respiratory disability	In Progress
Neuropsychiatric	RCT: Mindfulness-based online intervention [174]	China	Five Facets Mindfulness Questionnaire	In Progress
Neuropsychiatric Cardiovascular Respiratory	Prospective Cohort Exercise programs for patients with COVID or obstructive respiratory disease [175]	Germany	Participation limitations, quality of life, health status, fatigue, psychomental limitations and disorders, performance in different areas of life and ability to work	In Progress
Neuropsychiatric	RCT: REGAIN Trial [176]	UK	Health-related quality of life, dyspnea, cognitive function, health utility, physical activity participation, post-traumatic stress disorder symptom severity, depressive and anxiety symptoms	In Progress
General Inflammatory Response	Prospective cohort: Fit-COVID-19 Study [177]	Brazil	Immunomodulatory responses, e.g., cytokine level, peripheral blood mononuclear cells expression, physical activity level, cardiovascular and pulmonary function, respiratory muscle strength, functional exercise capacity	In Progress
Cardiovascular Respiratory Musculoskeletal	RCT: Rehabilitation Program for ICU patients [178]	Portugal	6-Minute walking test at 4 weeks and 12 weeks after discharge, and 12-item Short Survey at 12 weeks of FU	In progress

RCT: Randomized Controlled Trial. MCID: Minimal Clinically Important Difference.

## Data Availability

Not applicable.
